# Cognitive muscular therapy™ for knee osteoarthritis: A feasibility randomised controlled trial

**DOI:** 10.1016/j.ocarto.2026.100796

**Published:** 2026-04-01

**Authors:** Stephen J. Preece, Nathan Brookes, Jennifer Parker, Daniela Ghio, Amy Waghorne, Sally Gates, Caroline Fairhurst, Rebecca Wright, Anthony Jones, David Torgerson, Nicola E. Walsh

**Affiliations:** a Centre for Human Movement and Rehabilitation, School of Health and Society, University of Salford, Manchester, UK; b Division of Psychology and Mental Health, Faculty of Biology, School of Health Sciences, Medicine, and Health, University of Manchester, Manchester, UK; c York Trials Unit, University of York, York, UK; d Centre for Health and Clinical Research, University of the West of England, Bristol, UK; e Human Pain Research Group, Division of Neuroscience, University of Manchester, Manchester, UK

**Keywords:** Knee osteoarthritis, Physiotherapy, Rehabilitation, EMG, Biofeedback, Behavioural intervention

## Abstract

**Objective:**

People with knee osteoarthritis exhibit overactivity of the knee muscles during functional tasks. This will increase mechanical loads and may exacerbate pain. Cognitive Muscular Therapy™ (CMT) is a new conservative intervention that aims to reduce muscle overactivity and change habitual responses to pain. This study was designed to assess the feasibility of a future randomised controlled trial, designed to compare CMT with usual care.

**Methods:**

Patients with knee osteoarthritis, who had failed to benefit from previous therapeutic exercise, were randomised to receive CMT or usual care. Participants in the CMT arm were offered seven individual sessions, delivered by an NHS physiotherapist trained to deliver the intervention. Trial feasibility was assessed by monitoring recruitment, adherence, retention, treatment fidelity and acceptability through an embedded process evaluation. Secondary outcome measures included WOMAC and the Pain Catastrophizing Scale.

**Results:**

82 patients were recruited from 164 screened. Of the 42 allocated to the CMT arm, 32 completed the treatment. Retention was acceptable in the CMT arm but higher than anticipated in the usual care arm. Both patients and physiotherapists found the treatment to be acceptable, and the mean intervention fidelity score was 91%. Composite WOMAC score reduced by 17.1 points in the CMT arm from baseline to 20-weeks, and 2.8 points in the control arm over the same period.

**Conclusions:**

CMT is an acceptable intervention for people with knee osteoarthritis. Future large-scale trials are now required to quantify the clinical effectiveness of this promising new treatment.

**Trial registration:**

ISRCTN25291958.

## 1 Introduction

There is now a large body of evidence demonstrating that people with knee osteoarthritis (OA) display overactivity in the knee muscles during walking [1,2], standing [3] and other functional tasks [4]. Research has demonstrated the potentially damaging effect of this muscle overactivity, showing it will increase compressive loading [5], speed up the rate of cartilage loss [6] and increase the likelihood that patients will opt for a knee replacement at 5-year follow up [7]. Elevated muscle activity is likely to increase loading on the articular surface, the bone, joint capsule and periarticular structures, and could therefore increase nociceptive stimulation. Interventions that specifically target muscle overactivity may therefore reduce pain in knee OA [8].

We have developed a new intervention for knee OA, known as Cognitive Muscular Therapy™ (CMT). CMT integrates training to reduce overactivity of knee muscles and improve whole-body postural control within a framework of psychologically informed physiotherapy [[9], [10], [11]]. Rather than use simple muscle relaxation techniques, CMT specifically targets postural tone, the ongoing low-level muscle activity that supports the body against gravity [12]. Overactivity of the knee muscles is viewed as a compensatory biomechanical response to elevated flexor tone in the upper body. This therapeutic target is consistent with the observation of altered postural alignment in people with knee OA [[13], [14], [15]] and research linking trunk flexion with elevated activity of the knee flexor muscles [16,17]. With CMT, patients are taught to reduce flexor tone in the upper body, thereby reducing knee muscle overactivity.

CMT is markedly different from existing conservative interventions for knee OA. Although physiotherapy interventions have been developed that incorporate psychologically informed practice [18,19], these interventions typically integrate a focus on psychological factors within a muscle strengthening programme. In contrast, CMT adopts a multidimensional perspective, addressing the complex interaction between knee muscle overactivity, postural control, psychological factors [[20], [21], [22]] and central modulation of the pain experience [23,24]. Importantly, CMT teaches patients to become aware of how psychological factors can be linked to overactivity of the knee muscles, an idea which is consistent with recent research findings [[25], [26], [27]]. Another unique aspect of CMT is the use of EMG biofeedback to visualise of level of knee muscle activation. This enables patient to gain an experiential understanding of the link between knee muscle activity, postural control and thoughts about pain.

The aim of this study was to determine the feasibility of conducting a large-scale Randomised Controlled Trial (RCT), designed to compare CMT with usual care for people with knee OA who had failed to benefit from a muscle strengthening programme. Specifically, we investigated feasibility of recruitment, adherence, retention and feasibility of training National Health Service (NHS) physiotherapists to deliver CMT. There was also an embedded process evaluation designed to gain insight into patient and physiotherapist perceptions of the intervention via qualitative interviews. A secondary aim was to estimate the potential treatment effect.

## 2 Method

### 2.1 Study design and setting

This feasibility RCT was conducted between December 2021 and April 2024 with patients recruited through the United Kingdom (UK) NHS and social media (Facebook). Participating centres were three NHS secondary care centres in the Northwest of the UK. The study was approved by an NHS ethics committee (21/WM/0255) and by the University of Salford ethics committee. All participants provided written consent to participate, and all procedures were performed in accordance with the declaration of Helsinki.

### 2.2 Eligibility criteria

UK national guidelines [28] recommend programmes that integrate muscle strengthening with education as first line conservative management for knee OA. However, while existing exercise/education programmes are highly cost-effective, a large proportion of patients do not experience meaningful improvements in pain [29]. Given that the UK is a resource-limited healthcare setting, it was decided CMT would be unlikely to be offered on the NHS unless patients had previously tried, and failed to benefit from, an exercise/education programme that integrated some form of muscle strengthening. Therefore, to be included in the study, participants were required to have attended at least four muscle strengthening classes and to self-report an improvement in pain that was <15% from their pre-muscle strengthening condition.

In addition to the need to have tried muscle strengthening, participants were eligible if they were above 40 years of age, fluent in English and able to walk without an assistive device for at least 100 m. They were also required to have a clinical diagnosis of knee OA, assessed using the ACR criteria [30], and to have experienced knee OA pain for at least 6 months. Participants were excluded if they had dementia or any other cognitive impairment or a body mass index > 33 (as increased subcutaneous fat prevents collection of surface electromyography signals required for biofeedback). Participants were also excluded if they had previously had a lower limb arthroplasty, systemic inflammatory disorder or any significant balance disorder that could increase the risk of a fall.

### 2.3 Recruitment procedures

The original plan for recruitment was to identify people who had completed an ESCAPE-pain [31] class but failed to experience a 15% improvement in pain. However, this method proved infeasible as ESCAPE providers were not willing to share study materials in advance of the ESCAPE programme and data protection practices meant that it was not possible to identify participants after completion of the ESCAPE programme. Participants were therefore identified through three NHS musculoskeletal clinical assessment and triage services in the Northwest of England. In the UK, patients are typically referred to this triage service (from their GP) once they have tried, but failed to benefit from, standard physiotherapy. This triage service is the gateway for onward referral onto orthopaedic management of chronic knee pain.

At each triage service, electronic records were screened to identify potentially eligible patients who were sent the participant information sheet. Potential participants then contacted the research coordinator who screened for eligibility over the phone. Those who were eligible returned a postal consent form along with a data access form that provided consent for the research team to view previous x-ray data. Recruitment at two of the three triage services was slightly lower than anticipated. Therefore, a social media advert was also used to reach potential participants.

### 2.4 Randomisation and allocation

Participants were randomly allocated 1:1 to the CMT or usual care arm. The randomisation was carried out six weeks before treatment was due to commence and stratified by site using variable block sizes. The allocation sequence was generated using Stata V17 by an independent statistician at York Trials Unit, University of York, who was not involved in recruitment. Once baseline assessments were complete for a participant, the trial manager/administrator contacted the independent statistician to ascertain the participant's allocation. The trial manager then contacted participants to schedule intervention sessions for those allocated to the receive CMT.

### 2.5 Interventions

This feasibility study was designed to inform planning of a future pragmatic trial. Therefore, participants in the control arm received usual care. For patients who have not benefited from NHS physiotherapy, usual care includes treatments such as pharmacological management, intra-articular steroid, and surgery [28]. These treatments were available to participants in each arm of the trial.

CMT for knee osteoarthritis comprises five components, summarised below. Electromyography (EMG) biofeedback is used in components 2–5 to provide a visual representation of muscle activity. For this study, we used the Biometrics EMG system (Newport, UK), with surface EMG electrodes placed over the lateral quadriceps muscle (vastus lateralis) at the start of each clinical session.

• Component 1: Understanding knee pain: The physiotherapist challenges beliefs about chronic knee pain, explaining that increased muscle tension will increase mechanical loading at the knee, perpetuating pain. Pain neuroscience education is used to explain the idea that brain processing will modulate the pain experience.
• Component 2: General relaxation: The physiotherapist teaches awareness of muscular tension in lying/sitting positions. Using specific clinical techniques along with EMG biofeedback, patients are taught to fully relax the quadriceps.
• Component 3: Postural deconstruction: This component focuses on postural tone (low-level muscle activity that supports the body against gravity [12]). The aim is to reduce elevated flexor tone in upper body that may be triggered by factors related to a sedentary lifestyle. Altered postural control is linked to compensatory increases in knee muscle activation in standing and during functional movement [16,17]. Therefore, using EMG biofeedback supplemented with hands-on guidance, the physiotherapist teaches the patient to consciously reduce flexor tone in the upper body and associated compensatory overactivity of the knee muscles.
• Component 4: Contextual triggers: This component aims to teach patients to become aware of increased muscular contraction, which may relate to pain, pain expectations or pain-related beliefs. Using hands-on guidance and EMG biofeedback, patients gain experience of initiating pain-provoking movements, such as ascending stairs, with less overactivity of the knee muscles.
• Component 5: Functional integration: This component brings together learning across the first four components. Patients embed new muscle coordination patterns across functional tasks so that they become habitual.

The five components of CMT are delivered across seven individual physiotherapy sessions, lasting 1 h, typically every two weeks. Within each session, the physiotherapist follows a clearly delineated protocol, comprising 5–10 stages. Between supervised sessions, patients are provided with access to an online platform that provides material to support intervention delivery, such as animated videos. An example video can be viewed here: http://www.cogmustherapy.com/BMC_example_2. Further details of CMT are described in our intervention development paper [9]. Note that the name of components 1, 4 and 5 have been changed since this original publication.

Following the COVID-19 pandemic, there was limited capacity to deliver the research treatments at NHS physiotherapy outpatient clinics. Therefore, the intervention was delivered at three community sites, each located close to the recruiting site. Three NHS physiotherapists were trained to deliver the intervention. This training package was developed as part of a previous study [11] and involved 16 h of online learning along with two face-to-face training days. Each of the physiotherapists was experienced in treating chronic musculoskeletal pain (>5 years) and two had no prior experience of CMT. The third physiotherapist had taken part in an earlier training development study, delivering the intervention to two patients under observation from the lead physiotherapist (NB).

### 2.6 Assessment of intervention fidelity

An intervention fidelity checklist was developed with 25 items, each scored 0–3. Each item was used to quantify the physiotherapist's competency at delivering a specific stage of the protocol. Video recordings of 14 clinical sessions (two for each of the seven treatment sessions) were used to score intervention fidelity for each of the three physiotherapists. An average score for each physiotherapist was then obtained and normalised from 0 (low competency) to 100 (high competency).

### 2.7 Outcome measures

Clinical outcomes were collected by post or online form at baseline (prior to randomisation), 20 weeks post-randomisation and eight months post-randomisation. The 20-week time point typically coincided with the week after the final intervention session for participants in the CMT arm. The clinical outcome measures were: WOMAC Pain (range: 0–20; higher scores indicate greater pain) and WOMAC Composite (range: 0–96; higher scores indicate worse symptoms and functional limitation) [32]; Pain Catastrophizing Scale (range: 0–52; higher scores reflect greater pain-related catastrophizing) [33]; Tampa Scale of Kinesiophobia (range: 17–68; higher scores indicate greater fear of movement) [34]; Generalized Anxiety Disorder Scale, GAD-7 (range: 0–21; higher scores indicate more severe anxiety) [35]; and Patient Health Questionnaire, PHQ-9 (range: 0–27; higher scores indicate more severe depressive symptoms) [36]. In addition, data were collected on quality of life using the EQ-5D-5L [37], capacity to work using the work productivity and activity impairment (WPAI) questionnaire [38] and healthcare resource utilisation using a custom questionnaire. Finally, demographic data on age, gender and body mass index were collected at baseline and, if participants provided permission to access previous x-ray data, KL grade was also recorded.

### 2.8 Sample size

Sample sizes of between 24 and 70 have been recommended for feasibility trials [39,40]. Our aim was to recruit 90 participants to ensure at least 72 participants in the final analysis, allowing for a 20% attrition rate.

### 2.9 Feasibility criteria and statistical analysis

Baseline demographic and outcome data were summarised overall and by randomised group and recruitment and retention rates also summarised descriptively. For each outcome and group, the proportion of missing data was described and the outcome at each follow-up point summarised using mean and standard deviations. Changes from baseline were also summarised at each time point and effect sizes (Cohen's d) calculated along with 95% confidence intervals. Effect sizes of 0.2 were interpreted as small, 0.5 as medium and >0.8 as large [41]. To understand the feasibility of delivering a future large-scale trial, we defined a set of progression criteria based around recruitment, adherence, retention, acceptability to patients and feasibility of training physiotherapists (Table 1).

**Table 1:** Feasibility progression criteria.

Domain	Assessed by	Criteria		
		Red	Amber	Green
Recruitment	Average participants per site per month	<1.5	1.5–2.4	>2.4
Adherence	Number of participants attending 5 out of 7 clinical sessions in the CMT arm	<60%	60–80%	>80%
Trial retention	Participants providing 8-month outcome data	<60%	60–80%	>80%
Feasibility of training NHS physiotherapists to deliver the CMT intervention	Intervention fidelity score	<80%	80–90%	>90%
Acceptability to patients	Qualitative evaluation	–	–	–

### 2.10 Process evaluation

Semi-structured interviews were used to explore patient and physiotherapist perceptions of the intervention after the delivery/completion of CMT. These interviews were carried out by AW and SG via videoconference using a topic guide (see appendix 1) that was informed by acceptability of theoretical framework of acceptability for healthcare interventions [42]. A framework analysis [43] allowed for a combination of inductive and deductive analysis that was completed with the data collected from eight patients and three physiotherapists. Data from both groups were analysed together but consecutively. AW read through the transcripts and organised the data according to the framework. In data analysis sessions with DG, patterns across the data were developed to understand acceptability and areas for optimisation of the intervention.

## 3 Results

**Fig. 1:**
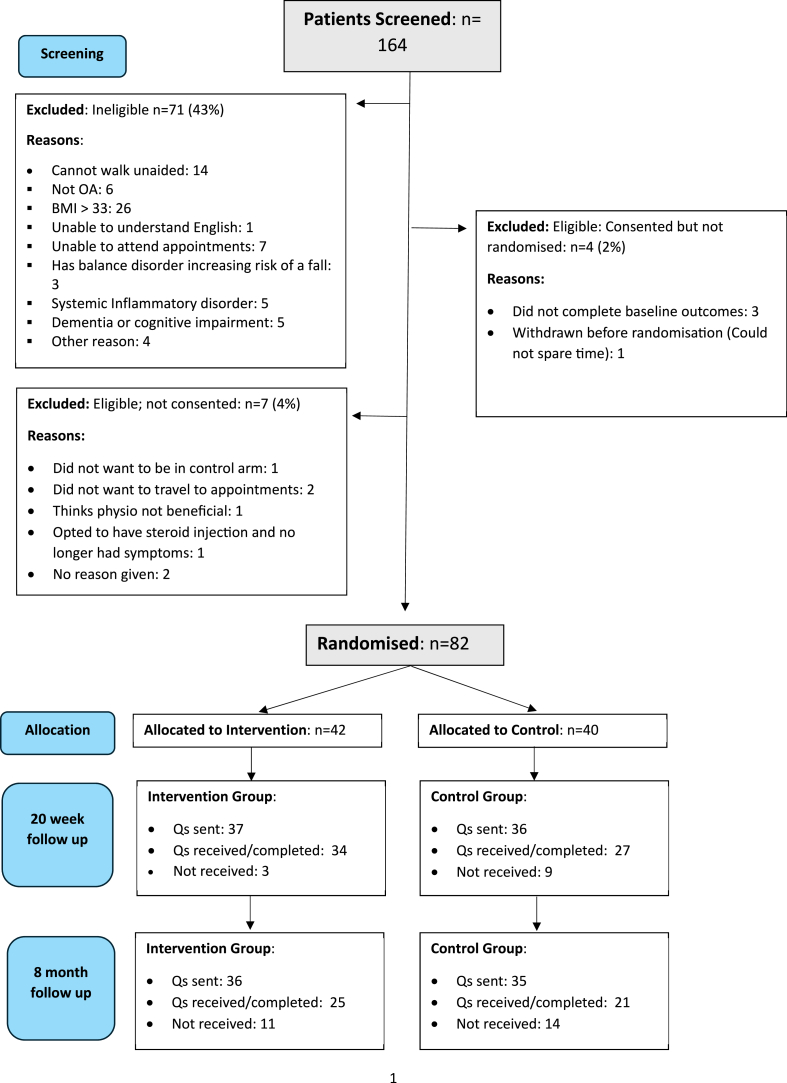
CONSORT diagram to show the flow of participants through the trial.

The flow of participants through the trial is shown in a CONSORT diagram (Fig. 1). Screening and recruitment began in September 2022 and completed in June 2023. Letters of invitation were sent to 1028 patients across the three recruitment sites, and a social media advert ran at two of the three sites for two weeks. In total, 164 patients responded, of which 29 (18%) were from social media. Of those who responded, 71 (43%) were found to be ineligible (Fig. 1), the main reason being BMI>33 or unable to walk unaided, with a further seven withdrawing before consent and four immediately after consent. The remaining 82 patients were randomised to the control (n = 40) or CMT arm (n = 42). Over the 10-month period of recruitment, an average of 5.5 patients were screened per site each month, which translated into an overall recruitment rate of 2.7 patients per site per month.

The two groups were balanced at baseline across of the demographic measures. Radiographic data were available for 43 of the 82 participants, with the majority (70%) having a KL grade of 3 or 4 indicating that they were at an advanced stage of the disease. The two groups were also balanced at baseline for most of the outcomes. However, WOMAC composite was slightly higher in the control group and GAD-7 and PHQ-9 were slightly higher in the CMT group (Table 2). For brevity, data on QoL, WPAI and healthcare utilisation have been presented in Appendix 2.

**Table 2:** Baseline demographic characteristics and baseline health outcomes. Mean (SD) are given unless stated otherwise.

Baseline characteristics	CMT (n = 42)	Control (n = 40)	Total (n = 82)
Age (years)	66.4 (8.8)	66.6 (9.0)	66.5 (8.9)
Gender: Females, n (%)	19 (45)	18 (45)	37 (45)
BMI (Kg/m^2^)	27.4 (3.1)	28.6 (2.8)	28.0 (2.8)
KL grade^a^, n (%)	N = 25	N = 18	N = 43
1	1 (4)	2 (11)	3 (7)
2	6 (24)	4 (22)	10 (23)
3	13 (52)	8 (44)	21 (49)
4	5 (20)	4 (22)	9 (21)
WOMAC pain^b^	8.5 (4.3)	8.9 (3.5)	8.7 (3.9)
WOMAC composite^b^	39.4 (18.7)	43.3 (16.6)	41.3 (17.8)
Pain catastrophizing scale	12.4 (12.5)	12.2 (11.4)	12.3 (11.9)
Tampa scale of kinesiophobia	39.9 (8.4)	40.3 (6.0)	40.1 (7.3)
GAD-7	4.0 (5.5)	3.4 (4.4)	3.7 (5.0)
PHQ-9	4.6 (5.4)	3.8 (3.4)	4.2 (4.5)

a Where participants provided permission to access previous x-ray data.

b Due to an error during data collection, WOMAC pain/composite are only available for n = 41 in the CMT group. (WOMAC = Western Ontario and McMaster Universities Osteoarthritis Index; GAD-7 = Generalized Anxiety Disorder Scale; PHQ-9 = Patient Health Questionnaire).

Of the 42 participants allocated to the CMT arm, a total of 32 (76%) completed the CMT treatment (defined as attending a minimum of five of the seven clinical sessions). Two patients did not formally withdraw but failed to attend any treatment sessions and one patient was found to be ineligible at the initial treatment session. Seven patients stopped attending treatment citing one of the following reasons: called up for a knee replacement, knee injury (unrelated to the intervention), health reasons other than knee, not disclosed and could not spare the time. Intervention fidelity scores for the three physiotherapists were 92%, 89% and 93% with a mean fidelity score of 91%. No adverse events were reported.

Of the 42 participants allocated the intervention arm, 34 (81%) returned outcomes at 20 weeks. This included all 32 participants who completed the CMT treatment. In the control group, 27 (68%) returned outcomes. Of the 13 control participants who did not return outcomes, four patients formally withdrew before 20-weeks citing one of the following reasons: could not spare the time, did not want to complete questionnaires, did not want to be in the control arm and called up for knee replacement. Nine patients in the control group were lost to follow-up. At 8 months, 25 participants (60%) in the CMT arm and 21 (53%) participants in the control arm returned outcomes. This equated to an overall trial retention at 8 months of 56%.

There were large changes in the WOMAC composite and WOMAC pain scores in the CMT group (Table 3, Table 4, Table 5). Specifically, between baseline and 20 weeks, the WOMAC composite score reduced by 17.1 points in the CMT group, compared to a reduction of 2.8 in the control group. When the two participants who did not complete the CMT intervention were excluded, the change in the WOMAC composite was 18.9 points, a within-group reduction of 49%. The large reductions in the WOMAC composite score in the CMT group appeared to be maintained at 8 months (Table 5). In contrast, there was an increase of 3 points in the control group, which led to a between group difference of 23.2 at 8-month follow-up. Changes in the Pain Catastrophizing Scale in the CMT group corresponded to medium effects at 20 weeks and small/medium effects at 8 months. Again, there were only small changes in the control group. There was very little change in the Tampa Scale of Kinesiophobia in either group at each time point. Interestingly, although there was a modest reduction in both the GAD-7 or PHQ-9 scores at 20 weeks in the CMT group, this change did not appear to be maintained at 8 months. The healthcare utilisation data suggested a trend towards more GP and orthopaedic consultations and higher medication usage in the control group (Appendix 2).

**Table 3:** Mean (SD) for each outcome at 20 weeks and 8 months post-randomisation for the two groups.

Outcome	20 weeks		8 months	
	CMT (n = 34)	Control (n = 27)	CMT (n = 25)	Control (n = 21)
WOMAC pain	4.5 (4.4)	7.9 (3.5)	3.8 (3.9)	8.7 (2.6)
WOMAC composite	20.5 (19.0)	38.4 (16.3)	19.0 (18.8)	42.2 (12.6)
Pain catastrophizing scale	5.9 (9.3)	10.8 (9.9)	6.5 (10.9)	13.4 (11.5)
Tampa scale of kinesiophobia	36.6 (7.3)	39.5 (6.7)	36.0 (9.0)	40.0 (6.7)
GAD-7	2.3 (3.7)	4.7 (5.6)	4.1 (6.1)	4.3 (3.3)
PHQ-9	2.7 (4.1)	4.4 (5.2)	4.0 (5.2)	4.6 (3.7)

**Table 4:** Within-group change in outcome measures from baseline to 20 weeks with effect sizes and 95% confidence intervals (CI).

Outcome	CMT (n = 34)			Control (n = 27)		
	Change	Effect size	95% CI	Change	Effect size	95% CI
WOMAC pain^a^	−3.6	−0.81	−5.6, −1.6	−0.6	−0.18	−1.8, 0.5
WOMAC composite^a^	−17.1	−0.92	−26.0, −8.3	−2.8	−0.16	−7.2, 1.7
Pain catastrophizing scale	−5.8	−0.51	−9.2, −2.4	−0.8	−0.08	−5.0, 3.5
Tampa scale of kinesiophobia	−0.4	−0.05	−4.2, 3.3	−0.3	−0.05	−3.5, 2.8
GAD-7	−1.6	−0.34	−3.0, −0.1	1.1	0.24	−0.8, 3.0
PHQ-9	−1.7	−0.36	−3.5, 0.0	0.7	0.17	−1.0, 2.5

a Due to an error during data collection, baseline WOMAC pain/composite are only available for n = 33 in the CMT group.

**Table 5:** Within-group change in outcome measures from baseline to 8 months with effect sizes and 95% confidence intervals (CI).

Outcome	CMT (n = 25)			Control (n = 21)		
	Change	Effect size	95% CI	Change	Effect size	95% CI
WOMAC pain^a^	−4.1	−0.99	−5.8, −2.3	0.7	0.25	−0.5, 2.0
WOMAC composite^a^	−18.6	−0.97	−25.6, −11.5	3.0	0.21	−3.0, 9.1
Pain catastrophizing scale	−5.1	−0.42	−8.1, −2.1	0.1	0.01	−4.7, 5.0
Tampa scale of kinesiophobia	−0.5	−0.05	−5.5, 4.6	−0.3	−0.05	−2.6, −2.1
GAD-7	−0.2	−0.03	−1.44, 1.04	1.4	0.46	−0.10, 2.86
PHQ-9	−1.0	−0.18	−26, 0.6	1.2	0.35	−0.5, 2.8

a Due to an error during data collection, baseline WOMAC pain/composite are only available for n = 24 in the CMT group.

In general patients found CMT acceptable, useful, and easy to engage with due to the sustainability of techniques: *“I'm still practicing, yes, what I learnt … Maybe for my lifetime”* (P229). They felt confident in completing exercises within their day and identified a perceived effectiveness in reducing negative beliefs and improving their ability to take part in daily activities: *“It's like having a new knee”* (P1). However, some participants found that intervention coherence was not always clear, as they felt CMT was complex and hard to define: *“Well, it's training your brain to think about … And it's all about breathing. So, I'm not very good at explaining it”* (P21), despite their engagement and desire to recommend CMT. Most participants did not find the intervention a burden, describing it as *“time well spent”* (P12), and wanted continued access to online materials after completion. The control group wanted access to more information to increase their knowledge of pain and the research and to help them to stay engaged in the study. Further information on each of the qualitative domains and corresponding themes is provided in Appendix 3, supplementary materials.

All three physiotherapists believed CMT had potential to be successful: *“It's got huge potential … I like the way it looks at the entire person”* (PT1). However, they struggled with delivery at the beginning because they were feeling apprehensive and unsure how to adapt protocols when facing resistance: *“It's just getting your head in the game with it”* (PT2). As they gained proficiency, confidence grew: *“By the end of the second session … I was getting a bit more used to it”* (PT2). They enjoyed the training but felt it was intense and needed more time for reflection: *“Could always do a little bit more face-to-face training”* (PT2). Time and effort required to deliver CMT was discussed as a challenge: *“It takes a lot more effort and a lot more patience”* (PT1), though this was not perceived negatively.

## 4 Discussion

This is the first randomised study designed to explore the potential of Cognitive Muscular Therapy™ to manage knee OA. The findings demonstrate that the intervention is acceptable to both patients and physiotherapists and that it would be feasible to conduct a large-scale RCT to compare CMT with usual care in a UK NHS setting. There were minimal changes in WOMAC scores in the control group but large reductions in the CMT group. The findings suggest that CMT may bring about sustained improvements in pain in people with knee OA who have failed to benefit from conventional physiotherapy. However, as this was a feasibility trial we did not conduct formal statistical testing to assess between-group treatment effects. A larger trial is now required to robustly quantify the clinical efficacy of this promising new intervention.

International guidelines recommend arthritis education and therapeutic exercise as first line management for knee OA [44]. However, a recent Cochrane review [29] concluded that the clinical benefits of exercise are of uncertain clinical importance. This idea is consistent with a study showing no statistical difference between best practice therapeutic exercise and placebo saline injection [45]. This placebo-controlled study observed within-group reductions of 10 and 7 points (on pain scale of 0–100) following exercise and placebo respectively. In contrast, we observed an 18-point (on pain scale of 0–100) reduction in pain in the CMT group at 20-week follow up. Furthermore, we observed a within-group change in the WOMAC composite score of 49% from baseline to 8-months. This is considerably larger than the difference of 17–22% that has been suggested as a within-group threshold for a minimally important rehabilitation effect [46]. While these are preliminary data, they suggest CMT may provide clinically meaningful improvement for patients with knee OA who do not respond to therapeutic exercise.

The CMT intervention was found to be acceptable to both patients and physiotherapists. Although 10 patients allocated to the CMT arm either withdrew or did not attend treatment, none cited any reasons related to the intervention. The process evaluation identified that, while patients were engaged with the treatment, they sometimes found it difficult to describe it to their friends and family. This may reflect the experiential learning integrated within CMT, specifically the ability to consciously regulate postural muscle tone, which is difficult to convey using everyday language. The three physiotherapists were also positive about CMT. Although they found the training and delivery challenging, with practice, they felt they gained confidence. In this trial, each physiotherapist delivered the treatment to an average of 10 patients. This is a relatively small number, and future trials could explore whether clinical outcomes improve as physiotherapists gain more practice of intervention delivery and become more familiar with the CMT protocols. Based on feedback from the physiotherapists, we made some minor improvements to our training package, such as more example videos of clinical delivery and improved animated videos to explaining biomechanical concepts.

This feasibility study was designed to inform planning of a larger RCT, and we looked specifically at five feasibility progression criteria (Table 1). We were able recruit >2.4 participants per site per month, demonstrating that recruitment was feasible. Furthermore, our qualitative work demonstrated that the intervention was acceptable to both patients and physiotherapists and our mean fidelity score (>90%) suggests our physiotherapist training is effective. However, while adherence to the intervention was good (76%), overall trial retention at 8 months was 56%. This would be considered unacceptable for a future RCT. Through our process evaluation, we identified the need to explain the importance of remaining in the trial and to continually communicate trial progress to participants. Financial incentives may also help to reduce attrition in future trials.

There are several limitations to this study that should be highlighted. Firstly, our screening criteria was based around a 15% threshold for improvement following therapeutic exercise. However, patients struggled to recall clinical benefit and therefore future studies might alternatively consider using an inclusion based around dissatisfaction with exercise. Secondly, this study was designed to inform planning of a pragmatic trial with a comparator of usual care. It is therefore not possible to gain insight into how CMT might perform in direct comparison with therapeutic exercise or to fully estimate placebo effects. Thirdly, we did not blind investigators to group allocation. However, as most outcomes were collected via an automated online system, this is unlikely to have impacted the findings. Finally, we had a larger-than-expected proportion of participants that were lost to follow up and have suggested measures to mitigate against this in future trials.

This study provides data showing that a future large-scale RCT comparing CMT with usual care is feasible within a UK NHS setting. The CMT intervention and study processes proved acceptable to patients and physiotherapists, and we have identified specific strategies for optimising processes for a future large-scale trial. If the findings of this feasibility trial are replicated in a large-scale RCT, then Cognitive Muscular Therapy™ has the potential to drive a change in the conservative management of knee OA.

## Contributions

SP, NB, DG, CF, JP, NW, AJ and DT contributed to the design of the study. NB, DG, AW, RW, CF, SG, JP and NW participated in data collection. SP, NB, JP and DG participated in the interpretation, analysis and discussion of data for the paper. SP drafted the first version of the manuscript. SP, NB, DG, JP, NW, RW, AJ and DT reviewed the first version of the manuscript and provided feedback. All authors read and approved the final version of the manuscript. SP takes overall responsibility for the integrity of the work as a whole, from inception to finished article.

## Role of the funding source

This study was funded by the UK National Institute for Health and Care Research (NIHR), Research for Patient Benefit programme, NIHR202203. The views expressed are those of the authors and not necessarily those of the NIHR or the Department of Health and Social Care. The NIHR played no role in the study design or implementation of the research.

## Competing interests

None.

## Figures and Tables

**Table:** 

Section 1: Background [for all]
Q1. Can you tell me what it’s like to have knee pain? Q2. Can you tell me about your experience of trial involvement?
**Section 2: General experiences of CMT intervention [only those in the treatment arm]**
Q3. I'm really interested in hearing about your experiences of using cognitive muscular therapy (CMT), can you tell me all about it? Q4. Can you tell me about anything you liked about CMT? [*probe specific components*] a. Can you tell me about anything that made it easy for you to use CMT? Q5. Can you tell me about anything you disliked about CMT? A. Can you tell me about anything that made it difficult for you to use CMT? Q6. [For those who stopped] can you tell me about what stopped you from using CMT? A. What are your thoughts about using CMT to manage pain? Q7. You had previously taken part in ESCAPE programme, can you tell me what that was like? A. How does CMT compare to ESCAPE programme?
**Section 3: Changes [only those in the treatment arm]**
Q8. Since using CMT, how do you feel about your knee pain now? Q9. Since using CMT, how do you feel about managing your knee pain? Q10. Can you tell me about anything that you feel has changed from using CMT? A. What has changed? *[Prompts: Anything different in your day-to-day life? The way you are managing your pain/your treatments/deal with things that can make your pain worse/deal with breathing/deal with stress?]* b. What do you think led to these changes?
**Section 4: Videos [only in the treatment arm]**
Q11. As part of the CMT you have the option to watch videos about the different topics. Can you remember whether you looked at any of the videos? a) How did you find them? *[Prompts: What did you find helpful/unhelpful?]* b) **[If didn't use]** can you tell me what made you decide not to look at any of the videos?
**Section 5: Trial procedures [for everyone who are part of the trial]**
Q12. I Am keen to hear about your experiences of taking part in research. What was it about the research that made you want to sign up? A. How did you feel about being put in groups? b. What did you enjoy/not enjoy from taking part in research? c. At the beginning of the research you needed to complete questionnaires, can you tell me can you tell me about how you found completing the questionnaires? [*Prompts: Can you tell me about any problems you came across when completing them? Can you tell me about any concerns you had?*]
**Section 6: Finish [for all]**
Q13. Do you have anything else you would like to tell me about your experiences of CMT that we haven't already covered?

**Table:** 

Section 1: Background
Q14. Can you tell me what it’s like to treat knee pain?
**Section 2: General experiences of delivering CMT intervention**
Q15. I'm really interested in hearing about your experiences of delivering cognitive muscular therapy (CMT), can you tell me all about it? A. How did you feel about the CMT? Q16. Can you tell me about anything you liked about CMT? [*probe specific components*] a. Can you tell me about anything that made it easy for you to deliver CMT? Q17. Can you tell me about anything you disliked about delivering CMT? A. Can you tell me about anything that made it difficult for you to deliver CMT? Q18. What are your thoughts about delivering CMT to manage pain? [*prompts about self-efficacy/confidence in delivery*]
**Section 3: Videos**
Q19. As part of the CMT you have the option to refer to videos about different topics, can you tell me what you thought of the videos? **a) [If didn't use]** can you tell me what made you decide not to look at any of the videos?
**Section 4: Finish**
Q20. Do you have anything else you would like to tell me about your experiences of delivering CMT that we haven't already covered?

**Table 4a:** Mean (SD) Work Productivity Activity Impairment (WPAI) data by treatment group at baseline

WPAI Items	Usual Care (N = 40)	CMT + Usual Care (N = 42)
Employed (%)	13 (32.5%)	12 (28.6%)
Hours missed from work due to knee OA among those employed (SD)	0.8 (2.2)	0.0 (0.0)
Hours missed from work due to holiday among those employed (SD)	0.1 (0.3)	2.7 (6.5)
Hours actually worked among those employed (SD)	23.0 (17.5)	28.3 (18.9)
Impact of knee on productivity among those employed (SD)	3.5 (3.4)	1.2 (2.1)
Impact of knee on ability (SD)	4.8 (2.5)	3.5 (2.8)
% Overall work impairment due to knee OA	8.8 (22.6)	3.2 (11.7)
% Activity impairment due to knee OA	48.1 (24.7)	35.1 (28.3)

**Table 4b:** Mean (SD) WPAI data by treatment group at 20 weeks

WPAI Items	Usual Care (N = 27)	CMT + Usual Care (N = 34)
Employed (%)	9 (33.3%)	8 (24.2%)
Hours missed from work due to knee OA among those employed (SD)	0.9 (2.7)	0.0 (0.0)
Hours missed from work due to holiday among those employed (SD)	1.0 (2.8)	2.0 (5.7)
Hours actually worked among those employed (SD)	32.6 (18.3)	28.6 (21.1)
Impact of knee on productivity among those employed (SD)	3.4 (2.9)	2.7 (3.7)
Impact of knee on ability (SD)	4.1 (3.1)	2.0 (2.8)
% Overall work impairment due to knee OA	10.7 (23.2)	5.9 (19.8)
% Activity impairment due to knee OA	40.7 (31.5)	20.0 (27.7)

**Table 4c:** Mean (SD) WPAI data by treatment group at 8 months

WPAI Items	Usual Care (N = 21)	CMT + Usual Care (N = 25)
Employed (%)	9 (42.9%)	5 (20%)
Hours missed from work due to knee OA among those employed (SD)	0.9 (2.7)	0.0 (0.0)
Hours missed from work due to holiday among those employed (SD)	2.8 (7.0)	0.0 (0.0)
Hours actually worked among those employed (SD)	25.5 (14.9)	33.5 (4.4)
Impact of knee on productivity among those employed (SD)	3.1 (3.3)	1.2 (1.6)
Impact of knee on ability (SD)	3.5 (2.6)	1.2 (1.7)
% Overall work impairment due to knee OA	12.5 (25.5)	2.5 (8.5)
% Activity impairment due to knee OA	35.5 (25.8)	11.8 (17.2)

**Table 5a:** Mean (SD) Healthcare resource utilisation (HRCU) by treatment group at baseline

HCRU	Usual care (N = 40)	CMT + usual care (N = 42)
**Knee-related care received as an NHS patient in primary care and the community**		
GP (doctor) at the GP practice (face-to-face)	0.3 (0.5)	0.9 (4.6)
GP (doctor) at the GP practice (online)	0.2 (0.4)	0.5 (2.5)
GP (doctor) at home (face-to-face)	0.0 (0.0)	0.0 (0.0)
GP (doctor) at home (online)	0.0 (0.0)	0.0 (0.0)
Physiotherapist (face-to-face)	1.3 (2.5)	1.8 (6.3)
Physiotherapist (online)	0.1 (0.3)	0.2 (0.6)
**Knee-related care received as an NHS patient in hospital**		
Orthopaedics outpatient (face-to-face)	0.3 (0.6)	0.5 (1.6)
Orthopaedics outpatient (online)	0.1 (0.2)	0.0 (0.0)
**Medications**		
Did the patient take medications for knee OA? (%)	31 (77.5)	29 (69.0)

**Table 5b:** Mean (SD) HCRU data by treatment group at 20 weeks

HCRU	Usual care (N = 27)	CMT + usual care (N = 34)
**Knee-related care received as an NHS patient in primary care and the community**		
GP (doctor) at the GP practice (face-to-face)	0.1 (0.3)	0.1 (0.2)
GP (doctor) at the GP practice (online)	0.3 (0.7)	0.1 (0.2)
GP (doctor) at home (face-to-face)	0.0 (0.0)	0.0 (0.0)
GP (doctor) at home (online)	0.0 (0.0)	0.0 (0.0)
Physiotherapist (face-to-face)	0.3 (0.8)	0.5 (2.3)
Physiotherapist (online)	0.1 (0.4)	0.0 (0.0)
**Knee-related care received as an NHS patient in hospital**		
Orthopaedics outpatient (face-to-face)	0.6 (1.1)	0.2 (0.7)
Orthopaedics outpatient (online)	0.0 (0.0)	0.1 (0.7)
**Medications**		
Did the patient take medications for knee OA? (%)	22 (81.5)	13 (38.2)

**Table 5c:** Mean (SD) HCRU by treatment group at 8 months

HCRU	Usual care (N = 21)	CMT + usual care (N = 25)
**Knee-related care received as an NHS patient in primary care and the community**		
GP (doctor) at the GP practice (face-to-face)	0.1 (0.5)	0.3 (1.2)
GP (doctor) at the GP practice (online)	0.1 (0.3)	0.1 (0.6)
GP (doctor) at home (face-to-face)	0.0 (0.0)	0.0 (0.0)
GP (doctor) at home (online)	0.0 (0.0)	0.0 (0.0)
Physiotherapist (face-to-face)	0.1 (0.3)	0.4 (1.8)
Physiotherapist (online)	0.0 (0.2)	0.2 (0.6)
**Knee-related care received as an NHS patient in hospital**		
Orthopaedics outpatient (face-to-face)	1.1 (2.3)	0.2 (0.5)
Orthopaedics outpatient (online)	0.0 (0.0)	0.1 (0.3)
**Medications**		
Did the patient take medications for knee OA? (%)	15 (71.4)	12 (48.0)

**Table 6a:** Mean (SD) EQ-5D-5L Utility (error with software used for data collection resulted in low completion)

Utility	Mean∗ (SD)	
	Usual care	CMT + usual care
Baseline	0.675 (0.163) n = 38	0.736 (0.145) n = 41
20-week	0.781 (0.102) n = 5∗	0.881 (0.085) n = 10∗
8 month	0.683 (0.225)n = 8∗	0.833 (0.162) n = 17∗

**Table 6b:** Mean (SD) EQ-5D-5L Quality Adjusted Life Years (error with software used for data collection resulted in low completion)

QALY	Mean∗ (SD)	
	Usual care	CMT + usual care
Baseline to 20-week	0.296 (0.038) n = 5	0.306 (0.032) n = 10
20-week to 8-month	0.297 (0.054) n = 3	0.329 (0.032) n = 7

**Table:** 

	Affective attitude *How individuals feel about the intervention*	Burden *The perceived effort required to ppt*	Ethicality *Degree it fits with individual's value*	Intervention Coherence*understand and how it works*	Opportunity Costs *Time/other benefits*	Perceived Effectiveness *Intervention will achieve intended purpose*	Self-efficacy *Confidence ppt have in their ability to perform intervention*	General Acceptability
Intervention	**Importance of therapeutic alliance** Building a relationship with physiotherapists, made to feel comfortable and CMT made sense. *“We seemed to click and got together and er I thoroughly enjoyed you know taking part in you know the exercises and the stuff she was asking me to do.”* P1 **Having to trust the process** Difficult to see how it will make sense, feeling awkward at first and noticing change, but having to accept there is no cure. *“at first I was a little bit sceptical thinking well, you know, the pain's there, it's not how I'm thinking about the pain … But then, you know, I realised the - so I have been doing things that maybe I wouldn't - you know, like the going downstairs, erm … more fluidly*.” P20	**Temperamental Technology** At times the technology was straightforward, but at times access to the videos did not work. *“Yeah. I don't - as you know my phone's a bit funny, so whether it was my phone, I've no idea. But I just - sometimes I was, you know, trying to do the exercises at home and I couldn't.”* P20 **Time well spent** Motivation was high, did not perceive the amount of time and effort needed as burden, because they were invested. *“I obviously sort of took it seriously and sort did it on a sort of regular basis and still now sort of reflect on it and probably not as much as I did during the programme”* P12	**Adjusting expectations around pain** expectations around cure/control and improved motivation to continue ***“****I've still got me knee pain … But, um, I was very happy, um. of things that I was told to do, and I still practice.”* P322 **Adjusting pain knowledge** Increased knowledge and understanding around pain and management **“***I suppose I've learned that there are ways of controlling pain … Other than by, erm, taking drugs, but I'm not really keen on taking drugs anyway, so - er, and I always knew that there were ways of controlling pain … It's just that I didn't know exactly how they worked.* P8	**Coherence not always clear** Hard to explain and concepts can be complex. *‘Well, it, it, it's, er, it's training- it's training your brain to think about … And it's all about breathing.’ So, I'm not very good at explaining it that way to other people*. P21 *I understood it, but I think it should be told more simpler … Whether it's just because I, whether it's just ‘cause I'm a simpleton, [laughing] I don't know* P322 **Other factors involved** Beyond just comprehension *“it was all right, what they were saying. You've just got to try. It's very difficult to change your ways, especially when you get 60, like me … It's very difficult to, to change what you've done all your life … But then, I suppose, if you want things to improve, then you have to.”* P313	**Travelling** Travel and parking to get to the sessions was an added task but benefit outweighed it generally *“Sometimes, I found it a little bit of hassle. Oh God, I've got to go all the way over to [place], and it takes so much out of my day … But with the outcome being what it is, it's been worth it”* P104 **Accommodating/ flexibility** Ppts appreciated flexibility from the team *“Erm, the venue was a bit of a pain, but that i- but it was … It just … They were very accommodating in, you know, changing times and things like that, so … We can't have everything. Erm, we can't have everything.”* P21	**Feeling positive about managing knee pain going forward** Recognising reduction in pain and planning on how to manage it in future *“I will just carry on doing … What I learned from CMT and that will be, hopefully be helpful … And don't have to have a knee operation.”* P229 **Significant decrease in knee pain** *“it's erm 90% disappeared. Erm and I don't think about it as much now. And if I do get a little bit of pain, then I think, well why have you done that? You know it's going to hurt if you do that, why are you doing it? Erm so it's just made me more aware of how to how to move around and what to do to minimise. It hasn't gone away totally, but I would say 90, 95% has. It's like it's like having a new knee”* P1 **Aspects that have improved** Other aspects beyond outcomes have improved *My home life's different. Erm, I'm, I'm not constantly moaning about knee pain! [Laughing] Erm, I'm able to play with the ki- my children a lot more. Erm, we went on holiday and, erm, for a long period of time in summer for six weeks, and last year I was unable to swim with them because my knees were so bad … This year I've been able to swim … So, people have noticed at school, erm, that, you know, I'm, I'm much more bubbly.* P21	**Confidence levels going beyond the intervention** *I'm quite confident to, er, to carry on, you know, trying to, trying to improve it … So I'd go to the nearest bench, then go to the next bench, then the next … I always had to go and then stop and I'm a little bit more, erm, confident now going, you know, somewhere where I don't know where the next bench is.P20* **Patience needed to finish** Need to see the intervention to the end and wait for the bigger picture *“to stay in the course because there's no point in starting it if I'm not going to finish. And erm so at the end of it yes I feel that it erm I gained from it.”* P4	**Sustainability of techniques** *“I'm still practicing, yes, what I learnt … Maybe for my lifetime, I think, with what I learnt, yeah. “*P229 **Focus on lessons learnt** Lessons learnt fit in with daily life and are incorporated in how to manage pain *“well, it does fit in, really, because I do, I do, it's on me mind all the time. I think I should relax my legs … So, it does fit in, really. And every time I go upstairs, I'm conscious of not holding on, and not, and doing it how I've been told to do it. And, you know, and standing up out of the chair, I'm conscious of that all the time. So, I, it does fit in.”* P313
Control	**Expectations and uncertainty** A lot of uncertainty and questions around pain, hoping that this would address it *I want, I wanted no more pain. And I thought, er, something like this, that they, they might have a, another drug, er, or something that I could have, that would help me with the pain, or would get rid of whatever's the matter with my knee. Because I've not found out what on earth is the matter with my knee.P19* **Disappointed in being in control group** *“Erm, initially er, I was not very impressed [laughing] … Erm, disappointed, disappointed … Let's say disappointed.”* P7	**Enjoyed process** Ppt were motivated to take part in the research *“Oh, good fun. Yeah, it was great. Yeah, people were nice and, erm, you know 2 h out of my life is not a lot. That's a few, you know it's - I'm retired, so it doesn't make any difference really. Erm, I found it very interesting. I found what they were doing that it - because at the end they could explain to me what it was all about.” P7* **Technology issue** *“Er, well, one of the, erm, surveys, I filled it in and I got to the end, but it, erm, summat, summat went pear-shaped … I don't know. I'm not, I'm not particularly good with, with technology. I mean, I can, kind of, get, get around it a bit, you know what I mean? “*P224	**Felt like they are giving back** Purpose provided motivation to take part in the research for the greater good *“In terms of the research, I, what, what I'd, I like is that, um, is, is the contribution. It, it's contributing to a, to a, you know, a bigger body of knowledge, really. And I, I just think, you know, every, everybody's, you know, got, got a, got a, a different story, as it were. And I, I just think, you know, fine, getting all that information directly from people, you know, just helps, or hopefully will help, you know, the, the next generation of people who come along, you know, who, you know, may have the same problems. So, you know, and, and, you know, the more knowledge we have, the, you know, the better the treatments, or the better the outcomes, so.”* P317	**Not exposed to the same information** It did not always feel fair as a cost to taking part. “*and the only thing I've done is fill in a couple of questionnaires and take this phone call. And that doesn't to me doesn't equal taking part in the study … Well being updated on how the study's going. Yep. Erm the results of the active trial. If people found it helpful, if you're going to try and roll it out, or do you see what I mean? Yep. Just really, information. I haven't had any I don't feel.”* P2	**Relevance around surveys** Completing surveys were not always easy to complete due to relevance. *“Erm. It was a pain in the neck. Well, some of the things I didn't think were relevant. And there was n-, no way that you could say that. Er. It comes back up and says, ‘You haven't completed. You haven't completed.’ Well, if you've, if you've not, if something isn't relevant, you can't complete it.”* P19	**Supportive of research – but wanted fairness** Ppt in the control group wanted more information and support – it was not effective being in control group. *“What they did was great. So, erm, I, I, the only thing I probably would have said was that if you say it at the outset, if you state it at the outset … that there will be two groups, one we w-, one will be controlled, I don't think that was really, erm, communicated to me. That was the only thing, that if it said, ‘Look, you may be in a control group, erm, but er, or you may be in the group that's being, erm, er, subject to the, erm, s-, whatever’ you know the … erm, what was going to happen. Erm, but at the end of it we will share with you, erm, the outcome and we'll tell you what's been done and things like that, erm, then that - I think that probably would have made it a little bit easier. So, the initial, er … I think what the main disappointment was that, erm, when I was informed that I was in the control group I was told that, erm, they wouldn't need to see me … er and that I would just fill in a questionnaire at the end of 20 weeks.”* P7 *“Probably the only thing that could have been done was to have a look at my knee [moderator chuckles] and see if they could see anything. Or come up with anything that was actually the matter.”* P19	**Having to manage pain through other means** Ppt discussed having to complete usual treatment pathways, such as medication, exercise *“And erm you know for balance, and also to sort of strengthen the knee. Lots of exercises to strengthen the muscles around the knee to take the pressure off the knee and I do a variety of them and I'd say I do all of them every day. Like sitting down doing exercises, just when we're round and about I'll do things. I've done the sitting down doing exercises thing erm for several weeks to get where I am and now I'm trying to maintain what I've got and also possibly improve slightly.”* P2	**More needed in communication** The role in the control group was unclear and so needed more for engagement and feeling part of the study *“Well for example you have the active participants in the trial and you've got the control group. Yep. I'm presuming that you've monitored the active participants over a number of weeks as to how their knee pain is etc. The control group you haven't really done that. We only had a couple of online questionnaires. OK. And perhaps an email. Perhaps equal erm equal er measuring of the two groups would be beneficial. OK. And in all honesty, I anticipated that's what I would get.”* P7
Physiotherapists	**Potential to be a successful intervention** *“I think it's got a, it's got huge potential. I like it, as I said before, I like the way it looks at the entire person. And particularly in this, the last few people that I've seen, the psychological, the awareness of belief and thoughts has become really obvious in this particular group of patients, how powerful that is to their progress and their success. So it's got, it's just got potential everywhere because the thoughts, beliefs, the fears, the predictions, that's the same no matter which person comes through the door.”* PT1 **Got better delivering over time** *“It's just getting your head in the game with it, really, so that's a little bit more challenging. Um. I suppose the more I did it, yeah, the easier it became.*” PT2	**Training** Liked the training and how well put together it was, enjoyed the face-to-face training. *“the face-to-face days were really, really good. Um, could always do a little, you know, a little bit more face-to-face training, I think, um, but that's probably just me and maybe how I learn to help. But, um, I think all the training was, was pretty good, and, and they did … “*PT2 **Overflow of information and time** A lot of information in the timeframe to digest, and took longer than expected. However, had the support needed *“It's in-depth, it's in detail, and the, the package is brilliant. […] The only issue I had with it, and it's all personal, is I can't take on that amount of information. I'm a practical movement-type person, so to hold all that information of what's happening when, I can't do it. I don't have the brain capacity to do that now.* PT1 *“I did go down on extra time and, and have a chat and go through some things with them. So, you know, there's not much more they could have done to help us, I don't think.”* PT2	**Trying to incorporate in current practice** *“Um, it certainly has made me, you know, think about it a bit more, and I am trying to bring it into my practice a little bit more, and especially choosing the patients, or maybe I can pick patients out who may be struggling doing the exercises. So I might kind of look at it in a different way and start with, uh, maybe how they're holding themselves, or if they are kind of really overactive in their quads, trying to initiate them to relax a little bit more, um, and understand, get them to understand their body a little bit more and, and relaxation and how important that is to kind of help to, um, offload the knee, really*.” PT2	**Something clicked** *“I understand it. It's, it just makes sense. And when you see it work and we see it make a difference, it just, it makes so much sense … It just makes sense, the whole, yeah, the whole concept just makes sense.”* PT1 **Topics found difficult and useful** *“Um, I suppose that's just going back to the contextual triggers that I found difficult, and the, the patients found tricky. Um, the stairs, I just don't think it replicated well … you know, back to their house. Um, but I liked the concept of it. It was just, it was hard to implement, and I don't think it kind of worked well with my patients.”* PT2	**Requires time and effort** *“But to get the quality right, and to be able to get the ideas across ‘cause the patients find it challenging ‘cause it's a, it's challenging all their previous thoughts and information they've been given, that takes a lot more effort and a lot more patience.”* PT1 *“it's spoilt me actually, to be honest, because it just feels a much better way of approaching things, but it's really difficult to deliver … in the clinical setting that I want to give it in. So it feels a bit bittersweet really.”* PT1 **Mentally more difficult than physically** *“- ‘Cause you know them better. Erm, but no, it wasn't, a-, apart from obviously having to get it in my head, that was the only, er, you know, it wasn't physically difficult. It was, I suppose it was mentally a bit more of a challenge*” PT3	**Variety of patient's responses** *“So I had w-, one person who literally took to it like a duck to water, didn't believe in his own ability to do it … but actually did brilliantly … And he was open to it. Had a, a guy who physically, I think, struggled with the whole [coughs] research ‘cause he didn't have any quads [sic] control [coughs] so he couldn't fine-control his knee. So that was difficult. […]* *- And then I had one who just completely shut down the psychology side of it, just totally shut it down, to the point was-, to the point one of her comments w-, was, ‘Right, we're gonna stop this now, we're gonna move on to the next bit.’ And the next bit actually was contextual triggers, so that was a challenging session. So she, she just put barriers to anything that was psychological, and I don't know …”* PT1 **Patients engagement** *“Positive because this makes sense. You can influence people's, not just their physical well-being, but their mental well-being and their well, generally, because of just doing the breathing stuff and identifying that. A lot of patients that I've seen have come back and they've used the breathing techniques in stressful environments, and generally found everything was so much nicer, and their movements and all sorts of things.”* PT1	**Confidence grows** *“Uh, obviously, I think that kind of grows the more you do it. So I was a little bit nervous at the start, um, and obviously through the first wave, um, everything's kind of new. The whole process is new, and each, like, session is quite new. I think by the end of the second session we were get … I was getting a bit more used to it, delivering it. Um, and then after, after wave two, I felt a bit more confident in myself, um, and how they might respond to it, or if there was any challenges, I'd know a bit more how to respond to them and help them. Um, I think it was still quite, oh, I always wanted to deliver it right, so I always kind of kept checking through my protocol and stuff like that to make sure I was following it.”* PT2	**Perceived implementation challenges** *“So we'd need … I, I would need a TV probably, you know, if we were gonna use the videos and things like that, then we'd need the equipment.”* P2 *“However, erm, I think-, yeah, if you, if you took a person from start to finish, a) you'd need a longer time because like at the minute I, my initial assessments are 5 min, and my follow-ups are half-an-hour. Well, you couldn't fit everything in that half-an-hour. So you know it would obviously, that would change entirely.”* PT3 **Practice has changed** *“Yeah. I mean I've taken the, the breathing assessments, the diaphragm breathing, the as-, like integrated breathing, the thoracic rotation stuff, and I've taken that into as many patients as I can in my usual cl-, practice. […]. I've just fine-tuned it and made it far more effective because of I know it, I've got more confidence. So that I've changed hugely. So yeah, it's changed a lot***.*”*** PT1 “i*t certainly has made me, you know, think about it a bit more, and I am trying to bring it into my practice a little bit more”* PT2

## Appendix 1 Semi-structured interview topic guide

The following topics/questions may be discussed. The qualitative work will remain flexible with respect to participants’ agendas but we will cover the broad topics/questions noted. It is common in qualitative work to iteratively develop topics and questions as new ideas emerge from early data collection. Therefore, we may add new topics as the interviews progress and data collection continues.

**Interview Questions for Patient Users**.

**Interview Questions for Physiotherapists Users**.
